# Regulation of Glucose Transporter Expression in Human Intestinal Caco-2 Cells following Exposure to an Anthocyanin-Rich Berry Extract

**DOI:** 10.1371/journal.pone.0078932

**Published:** 2013-11-13

**Authors:** Fawaz Alzaid, Hoi-Man Cheung, Victor R. Preedy, Paul A. Sharp

**Affiliations:** Diabetes and Nutritional Sciences Division, King's College London, London, United Kingdom; National Institute of Agronomic Research, France

## Abstract

Polyphenols contained within plant tissues are consumed in significant amounts in the human diet and are known to influence a number of biological processes. This study investigated the effects of an anthocyanin-rich berry-extract on glucose uptake by human intestinal Caco-2 cells. Acute exposure (15 min) to berry extract (0.125%, w/v) significantly decreased both sodium-dependent (Total uptake) and sodium-independent (facilitated uptake) ^3^H-D-glucose uptake. In longer-term studies, SGLT1 mRNA and GLUT2 mRNA expression were reduced significantly. Polyphenols are known to interact directly with glucose transporters to regulate the rate of glucose absorption. Our in vitro data support this mechanism and also suggest that berry flavonoids may modulate post-prandial glycaemia by decreasing glucose transporter expression. Further studies are warranted to investigate the longer term effects of berry flavonoids on the management of glycaemia in human volunteers.

## Introduction

The classical mechanism for intestinal glucose absorption involves uptake across the apical membrane of enterocytes via the sodium-dependent glucose transporter (SGLT1). Release of glucose into the circulation is mediated by the facilitated glucose transporter 2 (GLUT2) located on the basolateral surface of enterocytes. However, it has been recognised for a number of years that the apical uptake phase has two distinct elements: a saturable, phloridzin-sensitive (SGLT1) fraction and a diffusive component [1], suggesting that more than one transporter may be involved. Studies have now revealed that GLUT2 is also expressed at the apical membrane of enterocytes during the digestive phase and can contribute significantly to glucose absorption (reviewed in [2]), and may thus explain the diffusive uptake component.

Polyphenols, contained within plant tissues, are consumed in significant amounts in the human diet and are known to influence a number of biological processes including intestinal glucose absorption. Berries are rich in a wide range of bioactive polyphenolic compounds, particularly anthocyanins and other flavonoids [3]. Polyphenol-rich berry extracts have been shown to inhibit α-glucosidase activity *in vitro* [4], and to modify postprandial plasma glycaemia in healthy subjects following ingestion of sucrose [5]. Although acute inhibitory effects of dietary flavonoids on glucose uptake have been observed [6]–[8], little is known regarding their effects on the expression of glucose transporter genes. In this study we investigated the ability of an anthocyanin-rich berry extract to modulate glucose transporter mRNA expression, and glucose uptake by human intestinal Caco-2 cells.

## Materials and Methods

### Cell culture

Caco-2 TC7 cells [9], [10] (passages 40–45) were seeded into 6-well plates at a density of 40,000 cells per well and cultured for 19 d. Details of the maintenance of the cells and composition of cell culture medium have been previously described [6].

Cells were exposed for up to 16 h to a phenolic berry-extract, derived from blueberry, bilberry, cranberry, elderberry, raspberry seeds and strawberry (concentration range (0–0.25% (w/v); OptiBerry®, InterHealth Nutraceuticals, CA, USA). Approximately 60% (w/w) of the phenolic content of the extract is made up of anthocyanins (the anthocyanin content consists of: cyanidins 44.5%; delphinidins 26.1%; petunidins 14.4%; malvidins 8.9%). The extract does not contain free sugars. The extract was prepared in either serum-free cell culture medium (for chronic studies) or uptake buffer (for acute studies) and filter-sterilized before use.

### Glucose uptake assays

The acute and chronic effects of berry extract on glucose uptake (measured over 2 min) were assessed. For acute studies, Caco-2 cells were pre-equilibrated with berry extract for 15 min; [^3^H]-D-glucose (Perkin-Elmer, Bucks, UK) was added to initiate uptake. In chronic treatment studies cells were exposed to berry extract for 16 h. Treatment solutions were removed and the cells washed in uptake buffer. Fresh berry extract-free uptake buffer containing [^3^H]-D-glucose was added to initiate uptake. Experiments were performed using sodium-containing and sodium-free buffers. Uptake assays, analysis and buffer composition have been previously described [6]. Data were normalized to amounts of total protein/well, quantified using the protein quantification kit-rapid (Fluka, Dorset, UK).

### PCR

Total RNA was isolated from the cultured cells using TRIzol® (Invitrogen™ Life Technologies, Paisley, UK) according to the manufacturer's instructions. Following first strand cDNA synthesis, expression levels of glucose transporter mRNA and GAPDH mRNA (used as a housekeeping gene) were analysed by real-time quantitative PCR using an ABI Prism 7700HT Sequence Detection System and a Power SYBR® Green PCR master mix kit (Applied Biosystems™ Cheshire, UK). The primer sequences used for each gene are given in Supporting Information, Table S1. Quantitative measurements of glucose transporter relative to GAPDH gene expression were derived using the 2^−ΔΔCt^ method. Data have been normalised to the untreated control group in each experiment and are presented as the mean ± S.E.M.

### Western blotting

Caco-2 cell total protein was prepared as described previously [11]. Total proteins (40 μg) were subjected to SDS-PAGE. Following immobilization on nitrocellulose, the proteins were exposed to polyclonal anti-GLUT2 or anti-SGLT1 antibodies (1∶1000 dilution, Millipore, Consett, UK). Immunoreactivity was observed using a horseradish peroxidise-linked secondary antibody and Novex® ECL chemiluminescent substrate reagent kit (Invitrogen™ Life Technologies, Paisley UK). Band densities were measured using a densitometer (GS-800™) and Quantity One software (Bio-Rad Laboratories, Hertfordshire, UK). Actin protein levels were also measured (anti-actin antibody, 1∶2000 dilution, Sigma-Aldrich) to normalise for protein loading. Band densities were semi-quantified using a densitometer (GS-800™) and Quantity One software (Bio-Rad Laboratories, Hertfordshire, UK). Protein density data were expressed normalized to actin as the housekeeping protein.

### Statistics

All data are expressed as means ± SEM. Statistical analysis was carried out using SigmaPlot (version 12, Systat Software Inc. IL, USA). Student's unpaired t-test or one-way ANOVA followed by Dunnett's post-hoc test were used where appropriate to detect statistical differences (P<0.05) between control and test groups.

## Results

### Cell viability

There were no effects of berry extract treatment on Caco-2 cell viability, RNA yield or protein content (Supporting Information, Figure S1).

### Acute effects on glucose transport

**Figure 1:**
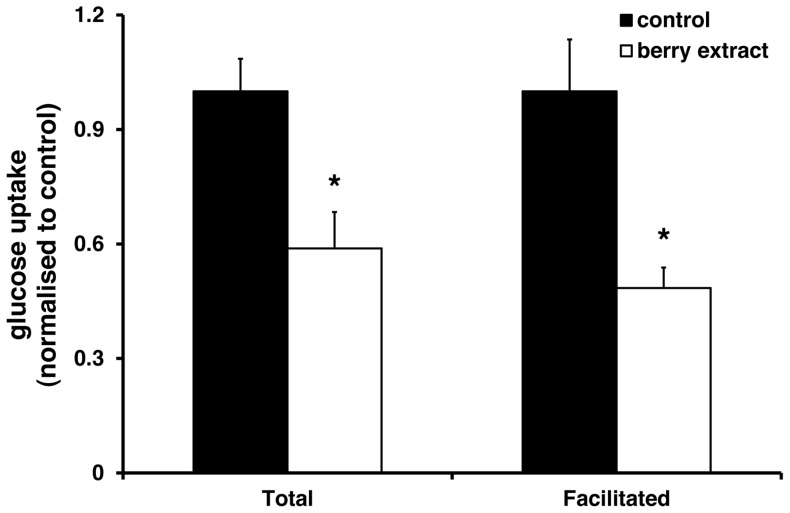
Acute effects of berry extract on glucose uptake. Caco-2 cells were treated acutely (15 min) with 0.125% (w/v) berry extract. Total glucose uptake (in Na^+^-containing buffer) and Facilitated (GLUT-mediated) uptake (in Na^+^-free buffer) (1 mM glucose) are presented as % control group. Data are the mean ± SEM (n = 11 in each group). *P<0.01, **P<0.04 (Student's unpaired t-test) compared with the respective control groups.

Single polyphenols can interact directly with glucose transporters to inhibit glucose uptake [6], [7]. Here, we investigated whether a more complex mixture of polyphenols, in the form of a berry extract, might also regulate intestinal glucose absorption. Total glucose uptake (measured in sodium-containing buffer; comprising both SGLT1 and GLUT-mediated uptake) and facilitated glucose uptake (measured in sodium-free buffer; which represents the GLUT-mediated component) were both significantly decreased after acute exposure to the berry extract (Figure 1). In our studies, the facilitated component accounted for approximately 58% of total glucose uptake (Total: 41.1±7.1 nmol/mg protein; Facilitated: 24.7±4.0 nmol/mg protein).

**Figure 2:**
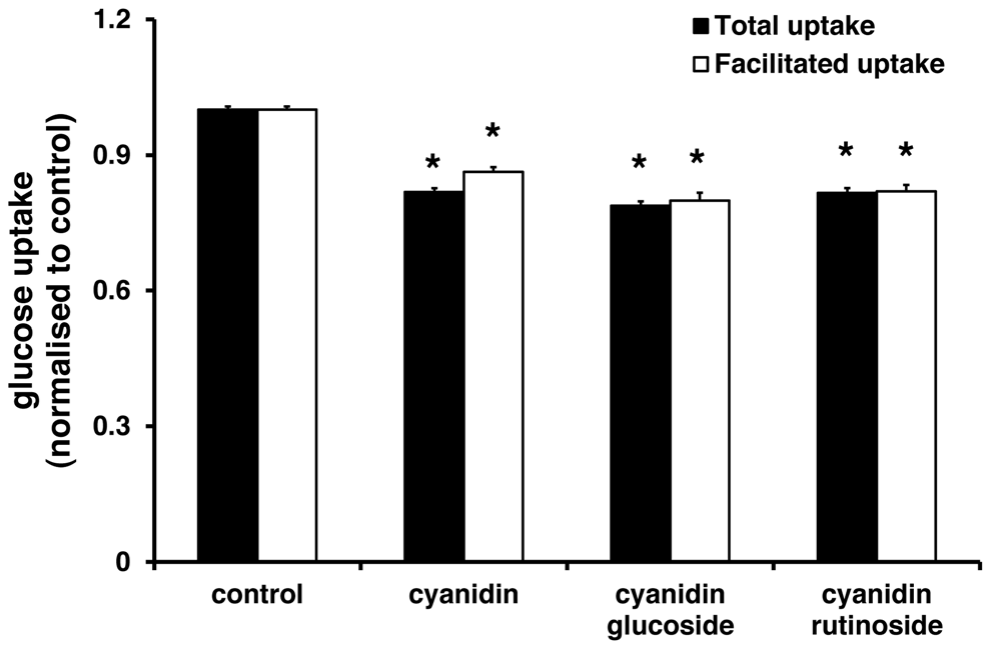
Acute effects of anthocyanins on glucose uptake. Caco-2 cells were treated acutely (15 min) with cyanidin aglycone, cyanidin-3-glucoside, or cyanidin-3-rutinoside (each 100 µM). Total and Facilitated glucose uptake (1 mM) are presented as % control group. Data are the mean ± SEM (n = 8 in each group). *P<0.01; one-way ANOVA followed by Dunnett's post-hoc test compared with the respective control groups.

The berry extract is likely to contain a range of polyphenols, present in their conjugated (largely glycosidated) and unconjugated (aglycone) forms, which may have differential effects on intestinal glucose transport. In the berry extract, more than 60% of the polyphenols present were anthocyanins, of which 44.5% comprised cyanidin-derivatives. Therefore, in parallel studies we tested the effects of cyanidin aglycone, cyanidin-3-glucoside, cyanidin-3-rutinoside on glucose uptake. All three compounds significantly inhibited both total and facilitated glucose uptake (Figure 2). In contrast, the model polyphenols phloretin (an aglycone) and phloridzin (a glucosidated form of phloretin), showed differential inhibition of glucose transport. Both compounds significantly decreased total glucose uptake; whereas only phloretin significantly decreased the facilitated uptake component (Supporting Information, Figure S2).

### Chronic effects on glucose transport

**Figure 3:**
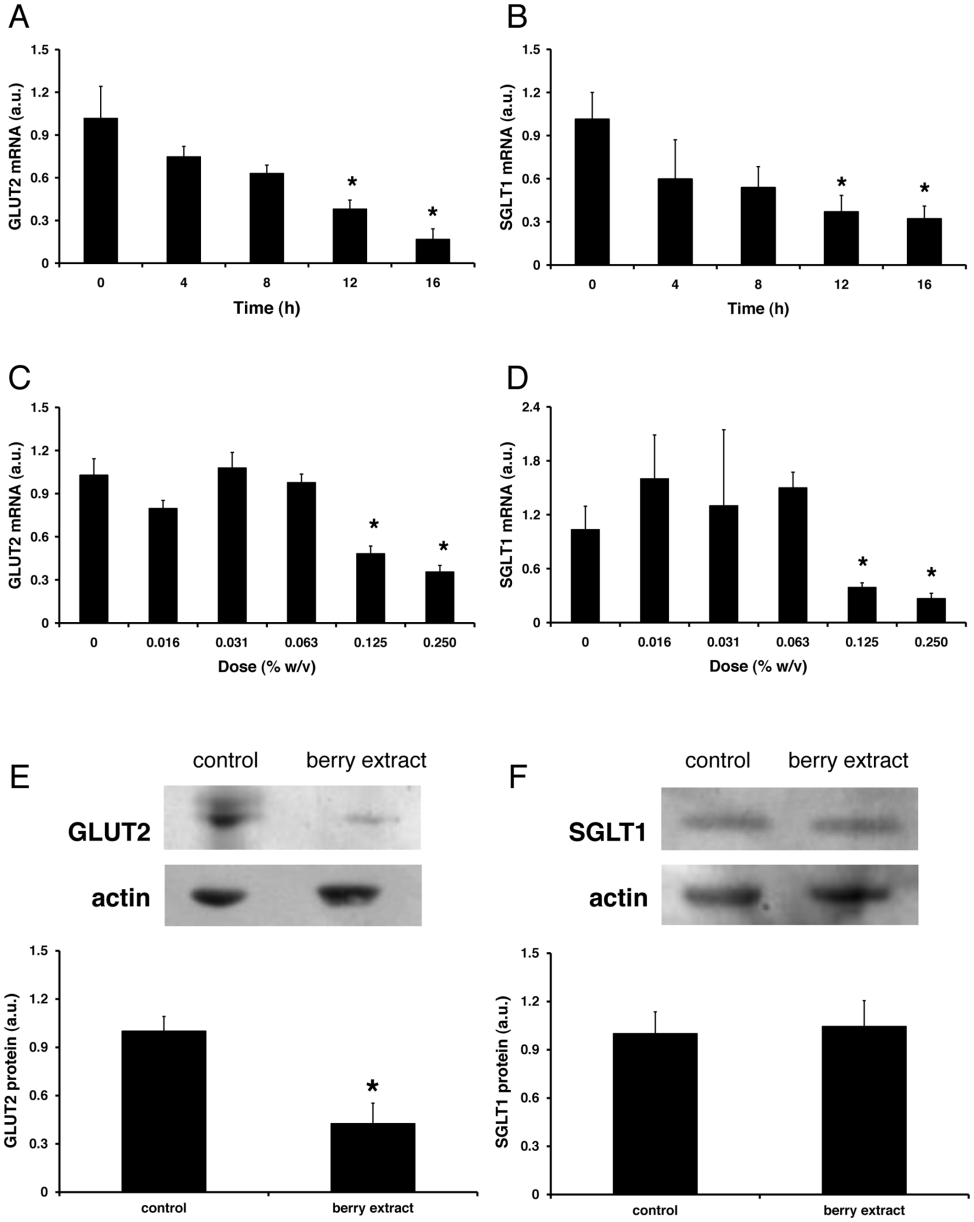
Effects of berry extract on glucose transporter expression. Caco-2 cells were treated with berry extract (0.125% (w/v)) for 0–16 h (A, B), or exposed to different concentrations of berry extract (0.016–0.25% (w/v)) for 16 h (C, D). GLUT2 mRNA (A, C) and SGLT1 mRNA (B, D) levels were normalised to GAPDH as housekeeping genes. Data are presented as mean (relative to the control group) ± SEM (n = 6). *P<0.05 (one-way ANOVA followed by Dunnett's post-hoc test) compared with the control group. Total protein was prepared from Caco-2 cells treated (16 h) with 0.125% (w/v) berry extract. Representative western blots and densitometric analysis are shown for GLUT2 (E) and SGLT1 (F). *P<0.05 (Student's unpaired t-test) compared with the control group.

Next we investigated the chronic effects on berry extract treatment on glucose transporter expression and glucose uptake in Caco-2 cells. GLUT2 mRNA was decreased by incubation with berry extract in a time- (Figure 3A) and dose-dependent manner (Figure 3C). Furthermore, GLUT2 protein levels were lower in cells exposed to berry extract (0.125% (w/v)) for 16 h (Figure 3E). There was also a time- and dose-dependent decrease in SGLT1 mRNA following exposure to berry extract (Figure 3B and 3D); however, in contrast to GLUT2 there was no difference in whole cell SGLT1 protein in berry-extract treated cells compare with untreated controls (Figure 3F).

To determine whether the inhibitory effect of the berry extract on GLUT2 and SGLT1 mRNA expression was specific to these transporters or part of a general inhibitory effect of gene transcription we also measured levels of other intestinal GLUTs, GLUT1 (a ubiquitously expressed glucose transporter) and GLUT5 (the intestinal fructose transporter). There was no significant effect of incubation with the berry extract on mRNA expression of either of these transporters (Supporting Information, Figure S3). In addition, we assessed the effect of the berry extract on the expression of genes implicated in intestinal glucose sensing; the sweet taste receptors T1R2 and T1R3 and the Cav1.3 calcium channel. T1R2 mRNA levels were significantly decreased following incubation with berry extract; however, there was no significant alteration in T1R3 or Cav1.3 mRNA levels in berry extract treated cells (Supporting Information, Figure S4).

**Figure 4:**
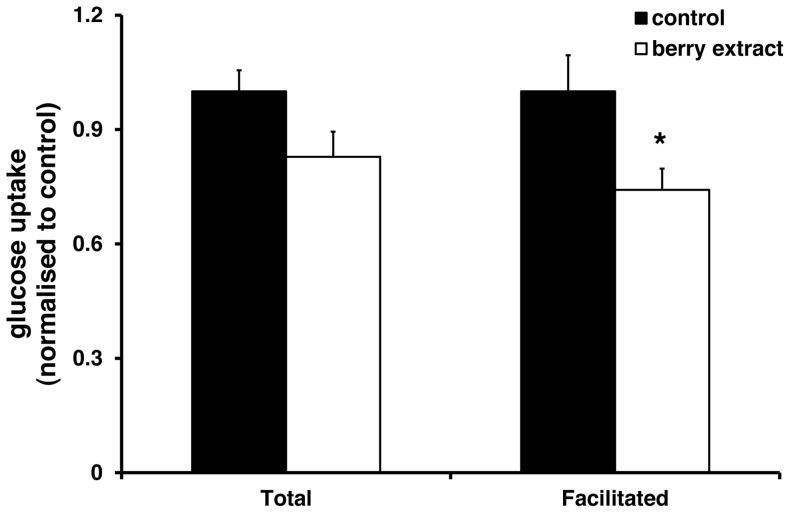
Chronic effects of berry extract on glucose uptake. Caco-2 cells were treated with 0.125% (w/v) berry extract for 16 h. Incubation medium was removed and replaced by fresh berry-free uptake medium. Total glucose uptake and Facilitated uptake are presented as the mean ± SEM (n = 11 in each group). *P<0.05 (Student's unpaired t-test) compared with the respective control groups.

Finally we investigated the longer term effects of the berry polyphenols on intestinal glucose uptake in Caco-2 cells. In these studies, the berry extract was washed-off prior to measuring glucose uptake. In contrast to the acute uptake studies, there was a small but statistically significant decrease in facilitated glucose uptake, but total glucose uptake was not significantly altered following exposure to berry extract (Figure 4).

## Discussion

Caco-2 cells are a widely utilized model to study intestinal nutrient transport and a number of studies have shown that polyphenolic compounds interact with glucose transporters in Caco-2 cells to limit glucose uptake [6]–[8], [12]. Here we report that treatment of Caco-2 cells with a berry extract, which contains a complex mixture of polyphenolic compounds, but is particularly rich in anthocyanins, also inhibited glucose uptake. Moreover, chronic exposure to the berry extract significantly decreased glucose transporter expression. The concentrations of berry extract used in our study (approximately 4 mmol/L at the highest dose, assuming an average molecular weight of 300 for individual phenolic components) are consistent with the levels of polyphenols that might be found in the intestinal lumen following consumption of polyphenol-rich beverages or fruits [13].

Evidence suggests that glucose absorption across the apical membrane of enterocytes is mediated by a combination of SGLT1 and GLUT2 [14], though the precise contribution of GLUT2 to lumenal glucose uptake remains controversial. Here, using the well-characterised Caco-2 cell model, we demonstrated the presence of two distinct glucose uptake pathways; one sodium-dependent, the other sodium-independent. Both total glucose uptake (measured in sodium-containing buffer; comprising both SGLT1- and GLUT-mediated uptake) and facilitated glucose uptake (measured in sodium-free buffer; which represents the GLUT-mediated component) were significantly decreased following acute exposure to the berry extract. Inhibition in this acute setting is likely to be due to physical interactions between berry polyphenols and the respective transporter proteins since there were no differences in transporter mRNA expression at this time. We [6] and others [7] have shown previously that polyphenols regulate glucose uptake in Caco-2 cells, with polyphenol-glucosides acting primarily on sodium-dependent transport while the corresponding aglycones regulate facilitated transport. These general findings were reproduced here using the model polyphenolics phloretin and phloridzin (the classical competitive SGLT1 inhibitor). However, for the cyanidins (the most abundant family of polyphenols in the berry extract), there were no differential effects of the aglycone, glucoside and rutinoside, respectively, on glucose uptake; all three compounds inhibited both total and facilitated uptake. This suggests that the inhibitory effects of the cyanidins on glucose uptake are non-specific and may occur due to steric hindrance rather than competitive inhibition.

Elevated intestinal glucose transporter levels have been reported in a number of diabetic animal models [15]–[17] and following prolonged feeding of high carbohydrate diets [18]–[21], and this contributes directly to hyperglycaemic status of these animal models. It follows therefore that compounds which regulate glucose transporter expression may be useful as anti-hyperglycaemic agents. We therefore investigated whether chronic exposure to berry extract might regulate glucose transporter expression in Caco-2 cells. Both SGLT1 and GLUT2 mRNA were significantly decreased following berry treatment suggesting potentially beneficial effects on the intestinal glucose transport machinery. In this context, previous work has shown that polyphenols from Yerba Maté decreased intestinal SGLT1 mRNA in alloxan-diabetic rats [22]. Interestingly, our data indicate that the effects of berry polyphenols on glucose transporter mRNA expression are limited to the major intestinal glucose transporters SGLT1 and GLUT2 and do not influence expression of the fructose transporter GLUT5 or the ubiquitous glucose transporter GLUT1. Despite significant decreases in GLUT2 and SGLT1 mRNA there was no significant effect of chronic berry extract treatment on total glucose uptake, but there was a small but significant decrease in the facilitated glucose uptake component. Taken together, our data suggest two potential modes of action by which berry polyphenols might regulate intestinal glucose transport: (a) physical interaction with the glucose transporters to regulate the rate of glucose uptake; (b) genomic modulation of transporter expression, influencing maximal transport capacity.

In keeping with the mRNA expression data, whole cell GLUT2 protein was significantly decreased by chronic exposure to berry polyphenols; however, there was no effect on whole cell SGLT1 protein levels. GLUT2 has been detected at both the apical membrane and basolateral membrane of Caco-2 cells [23] and there is evidence that intracellular pools of SGLT1 can be trafficked between the plasma membrane and cellular organelles [24]. In rat jejunal enterocytes, apical membrane levels of both transporters can alter rapidly in response cell signalling events [25], [26]. Further studies are therefore required to address the effect of berry polyphenols on cell signalling events and the cellular distribution of GLUT2 and SGLT1.

Apical GLUT2 levels are controlled largely by the prevailing lumenal glucose concentration, and are contingent on both an intact SGLT1-mediated glucose transport pathway [27]; calcium influx via the plasma membrane Cav1.3 channel [28]; and signalling via the T1R family of sweet taste receptors [29]. Published data suggest that an intact mucosa is required to sense changes in lumenal glucose concentration and modify the rate of glucose absorption accordingly [30] indicating that changes in glucose transport might be mediated via the release of gut hormones and/or neural input [21], [31]. This would support the notion that the sweet taste receptors T1R2 and T1R3 are localised to enteroendocrine cells [21]. However, other studies have identified sweet taste receptors in a number of intestinal cell types including enterocytes [29], suggesting that absorptive cells have the ability to sense changes in lumenal glucose directly via sweet taste receptor signalling and regulate the rate of absorption accordingly. Sweet taste receptor mRNA has also been detected in Caco-2 cells [32] and in our study we found that T1R2 mRNA expression was down-regulated following exposure to berry extract; however, there was no effect on T1R3 (or Cav1.3) expression.

It is suggested that apical GLUT2 is required for normal physiological intestinal glucose absorption [2], [14]. However, it is also likely that apical GLUT2 recruitment would be detrimental to health as it would result in an increased rate of intestinal glucose absorption, an enhanced glycaemic response to a meal, and the release of high levels of insulin into the circulation. In the long term these events are risk factors for obesity and type 2 diabetes. Thus strategies which modulate apical GLUT2 expression may benefit groups at risk of developing these metabolic disorders. Our *in vitro* findings suggest that dietary berry polyphenols can regulate GLUT2 expression and glucose uptake. Single meal studies in healthy human volunteers have shown that berry polyphenols modify the postprandial plasma glucose response to sucrose [5]. Further studies are therefore warranted to investigate the longer term effects of berry flavonoids on post-prandial glycaemia and glucose transporter expression in euglycaemic and hyperglycaemic human populations.

In summary, acute exposure to an anthocyanin-rich berry extract significantly decreased both Na^+^-dependent and Na^+^-independent glucose uptake in human intestinal Caco-2 cells. Longer-term exposure to berry extract significantly reduced GLUT2 mRNA and protein levels and resulted in a small but significant inhibition of the facilitated glucose uptake component. These findings provide further evidence that dietary polyphenols act as important modulators of the rate of intestinal glucose absorption.

## Supporting Information

Figure S1 **Effects of berry extract on Caco-2 cell viability, RNA content and protein content.** Caco-2 cells were treated with berry extract at 0.125% (w/v) for 16 h. (A) Cell viability was quantified with the Trypan blue dye-exclusion method. (B) RNA concentration in the extracts of Caco-2 cells was quantified with the NanoDrop spectrophotometer. (C) Protein concentration was measured using the Bradford assay method. Data is presented as mean ± SEM, n = 6.
(TIF)

Figure S2 **Acute effects polyphenolic treatments on glucose uptake.** Caco-2 cells were treated acutely (15 min) with model polyphenolic inhibitors of glucose transport phloretin or phloridzin (each 100 µM). Total glucose uptake (black bars) and Facilitated (GLUT-mediated) uptake (white bars) are presented as the mean ± SEM (n = 4 in each group). *P<0.001; one-way ANOVA followed by Dunnett's post-hoc test compared with the respective control groups.
(TIF)

Figure S3 **Effect of berry extract on GLUT1 and GLUT5 expression.** Caco-2 cells were treated with berry extract (0.125% (w/v)) for 16 h. Levels of GLUT1 (A) and GLUT5 (B) mRNA were normalised to GAPDH. Data are presented as the mean (relative to the control group) ± SEM (n = 4–6 in each group).
(TIF)

Figure S4 **Effect of berry extract on Cav1.3, T1R2 and T1R3 expression.** Caco-2 cells were treated with berry extract (0.125% (w/v)) for 16 h. Levels of the calcium channel Cav1.3 (A) and the sweet taste receptors T1R2 (B) and T1R3 (C) mRNA were normalised to GAPDH. Data are presented as the mean (relative to the control group) ± SEM (n = 4–6 in each group). *P<0.05, Student's unpaired t-test.
(TIF)

Table S1 **Primer sequences used for quantitative PCR.**
(TIF)
