# Supplementary figures and images for: Regulation of Glucose Transporter Expression in Human Intestinal Caco-2 Cells following Exposure to an Anthocyanin-Rich Berry Extract
Source: PLoS One. 2013 Nov 13;8(11):e78932. doi: 10.1371/journal.pone.0078932 (PMC3827299; doi:10.1371/journal.pone.0078932)

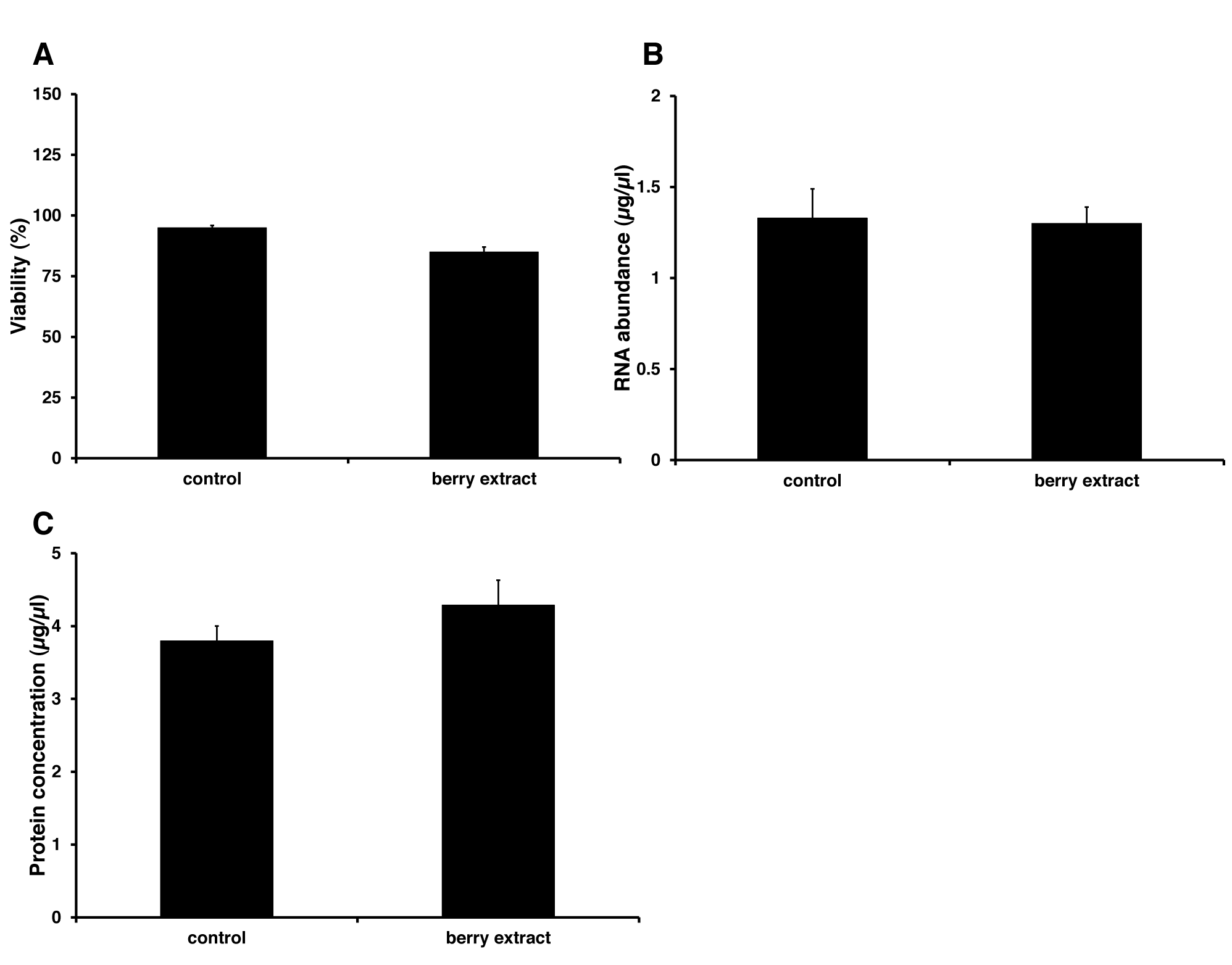

Supplement: Figure S1 — Effects of berry extract on Caco-2 cell viability, RNA content and protein content. Caco-2 cells were treated with berry extract at 0.125% (w/v) for 16 h. (A) Cell viability was quantified with the Trypan blue dye-exclusion method. (B) RNA concentration in the extracts of Caco-2 cells was quantified with the NanoDrop spectrophotometer. (C) Protein concentration was measured using the Bradford assay method. Data is presented as mean ± SEM, n = 6. (TIF) [file pone.0078932.s001.tif]

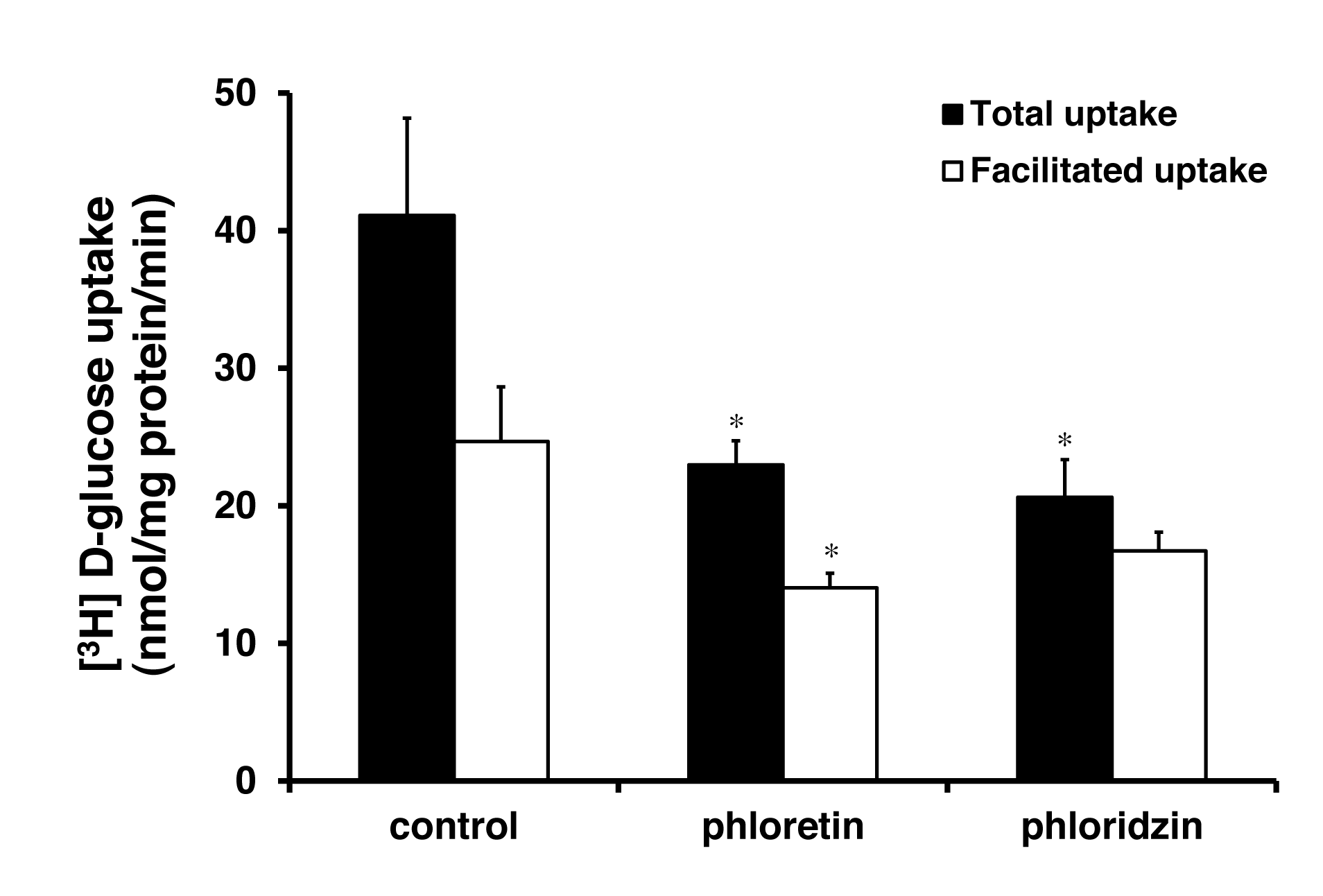

Supplement: Figure S2 — Acute effects polyphenolic treatments on glucose uptake. Caco-2 cells were treated acutely (15 min) with model polyphenolic inhibitors of glucose transport phloretin or phloridzin (each 100 µM). Total glucose uptake (black bars) and Facilitated (GLUT-mediated) uptake (white bars) are presented as the mean ± SEM (n = 4 in each group). *P<0.001; one-way ANOVA followed by Dunnett's post-hoc test compared with the respective control groups. (TIF) [file pone.0078932.s002.tif]

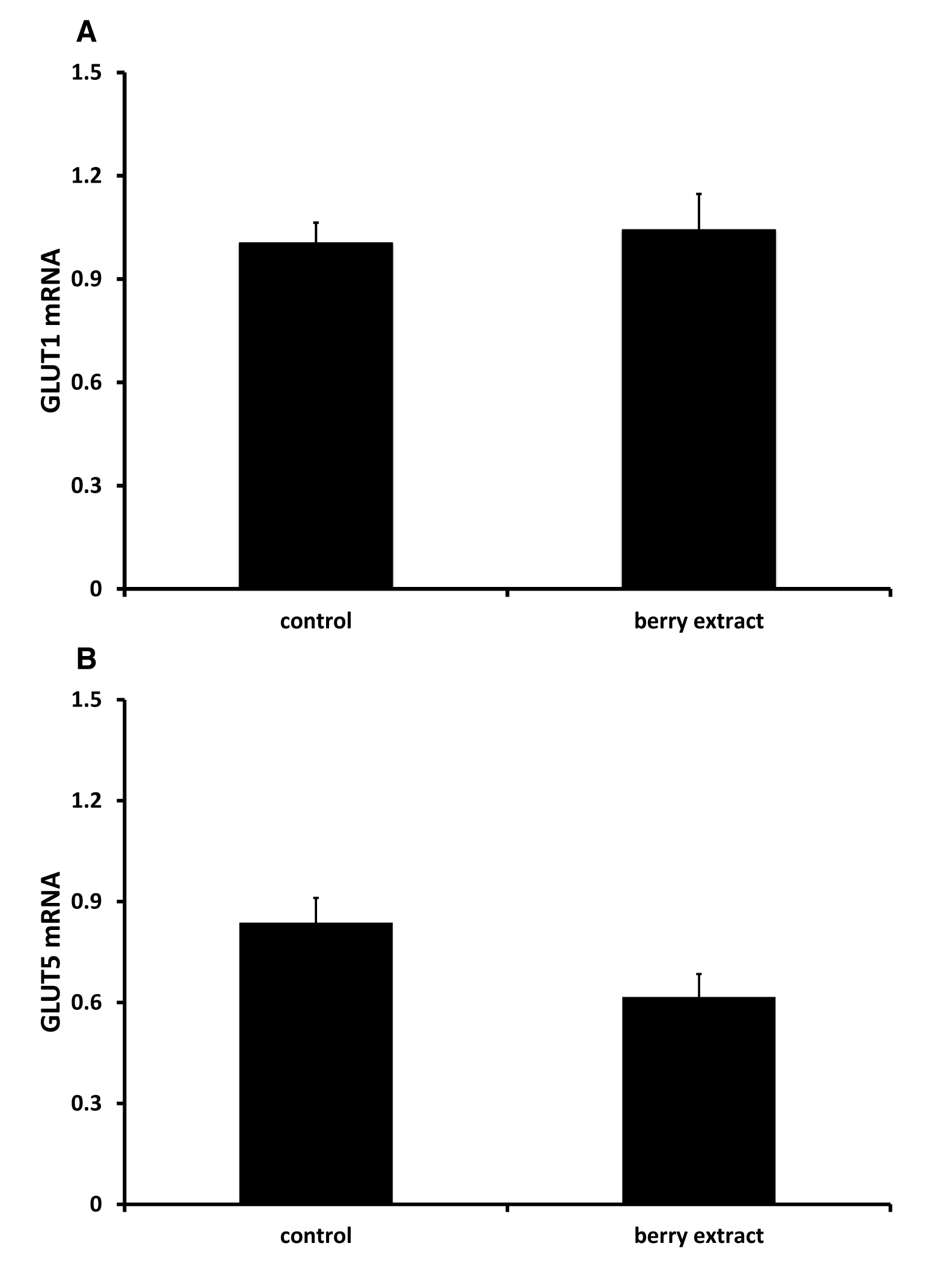

Supplement: Figure S3 — Effect of berry extract on GLUT1 and GLUT5 expression. Caco-2 cells were treated with berry extract (0.125% (w/v)) for 16 h. Levels of GLUT1 (A) and GLUT5 (B) mRNA were normalised to GAPDH. Data are presented as the mean (relative to the control group) ± SEM (n = 4–6 in each group). (TIF) [file pone.0078932.s003.tif]

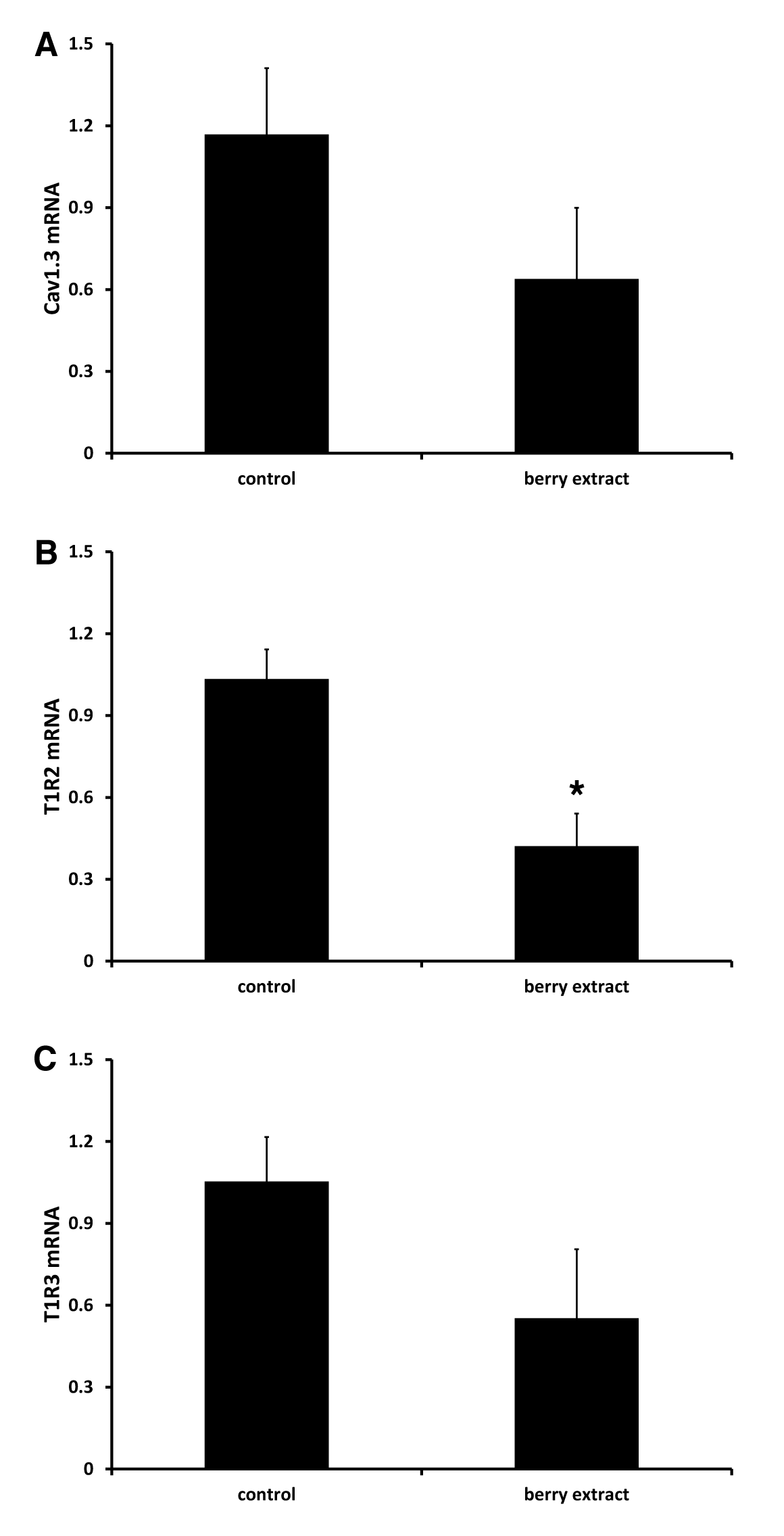

Supplement: Figure S4 — Effect of berry extract on Cav1.3, T1R2 and T1R3 expression. Caco-2 cells were treated with berry extract (0.125% (w/v)) for 16 h. Levels of the calcium channel Cav1.3 (A) and the sweet taste receptors T1R2 (B) and T1R3 (C) mRNA were normalised to GAPDH. Data are presented as the mean (relative to the control group) ± SEM (n = 4–6 in each group). *P<0.05, Student's unpaired t-test. (TIF) [file pone.0078932.s004.tif]

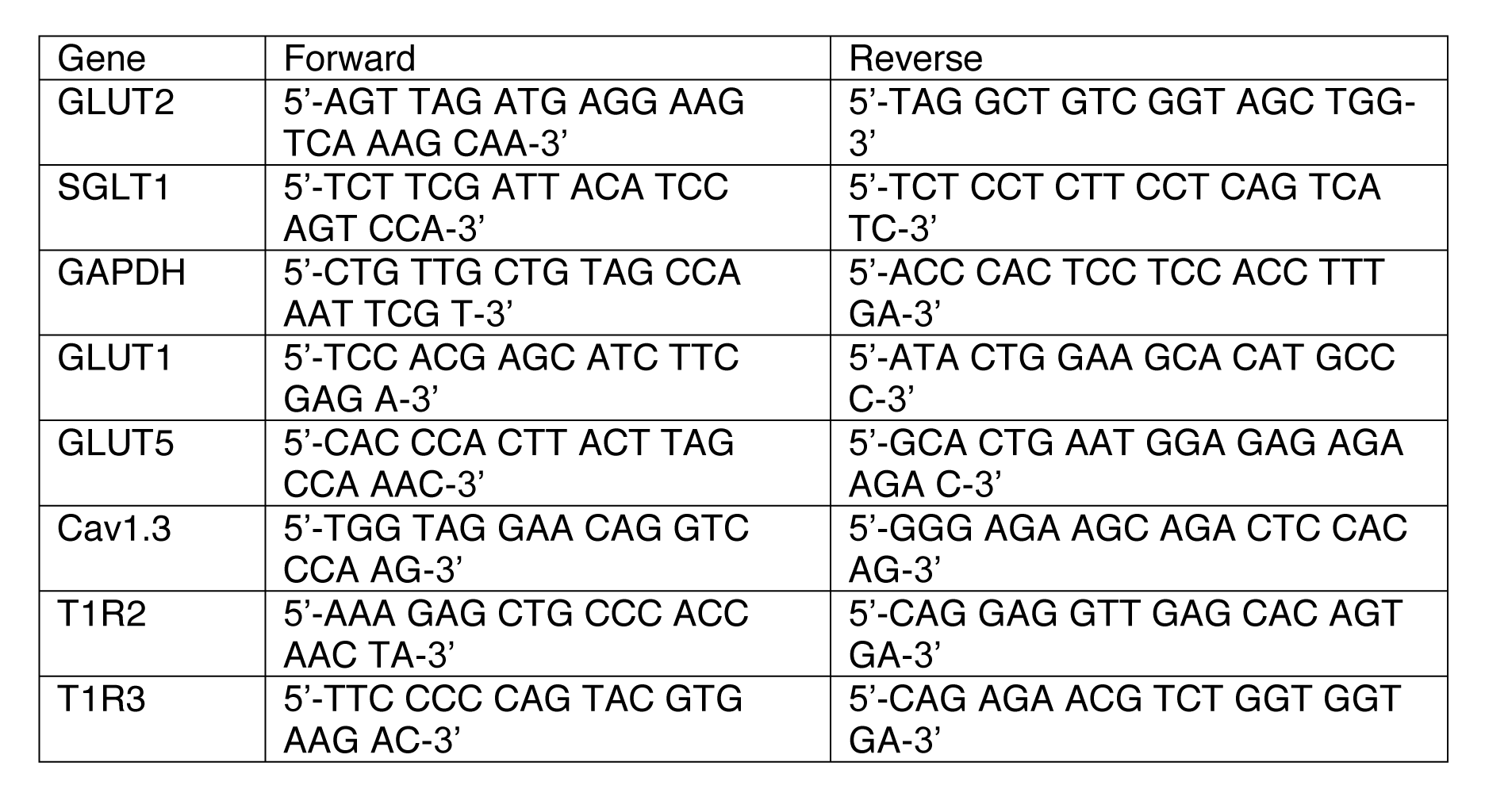

Supplement: Table S1 — Primer sequences used for quantitative PCR. (TIF) [file pone.0078932.s005.tif]
